# An epidemiologic study of physician-diagnosed chronic obstructive pulmonary disease in the Turkish population: COPDTURKEY-1

**DOI:** 10.3906/sag-1908-35

**Published:** 2020-02-13

**Authors:** Tarkan ÖZDEMİR, Nilgün YILMAZ DEMİRCİ, Hatice KILIÇ, Orhan KOÇ, Akın KAYA, Can ÖZTÜRK

**Affiliations:** 1 Department of Chest Diseases, University of Health Sciences, Dr. Abdurrahman Yurtaslan Ankara Oncology Research andTraining Hospital, Ankara Turkey; 2 Department of Chest Diseases, Faculty of Medicine, Gazi University, Ankara Turkey; 3 Department of Chest Diseases, School of Medicine, Yildirim Beyazit University, Ankara Turkey; 4 Department of Management, Social Security Institution, Ankara Turkey; 5 Department of Chest Diseases, Faculty of Medicine, Ankara University, Ankara Turkey

**Keywords:** Chronic obstructive pulmonary disease, epidemiology, population characteristics, prevalence, incidence, mortality, health information systems

## Abstract

**Background/aim:**

Chronic obstructive pulmonary disease (COPD) is a common disease characterized by persistent airflow limitation and respiratory symptoms. It is a leading cause of morbidity and mortality all over the world. Our data on COPD in Turkey are limited. This study was intended to examine the epidemiologic characteristics of COPD in the Turkish population, between 2012 and 2016.

**Materials and methods:**

This population-based, descriptive, surveillance study examined physician-diagnosed COPD prevalence, incidence, and mortality in Turkey. The database of the Social Security System of Turkey was scanned and ICD-10 J44.0-J44.9 codes for diagnostic and/or therapeutic purposes were evaluated retrospectively.

**Results:**

In 2016, there were 3,434,262 cases of COPD (56.2% men) in Turkey, and the mean age of patients was 61.62 ± 14.76 years. From 2012 to 2016, the annual overall prevalence rates of physician-diagnosed COPD rose from 4.3% to 5.8%, which was a 35.0% relative increase (P < 0.05). In women, this rate rose from 3.7% to 5.1% (38% increase), and in men, it rose from 4.9% to 6.7% (37% increase). During the study period, the overall incidence decreased from 8.5 per 1000 adults in 2012 to 6.3 per 1000 adults in 2016, representing a decrease of 26.6% (P < 0.001). The annual incidence rates of physician-diagnosed COPD decreased 25.4% in women and 27.9% in men. The overall mortality was 4.3% in 2012, and 4.2% in 2016. The mortality rate in women was 3.5% in 2012 and 3.7% in 2016, and 5% in 2012 and 4.7% in 2016 in men. The mean prevalence by region was 5.26% (range 3.79%–7.65%). The Black Sea region had the highest COPD prevalence.

**Conclusion:**

COPD is a very common and serious cause of morbidity and mortality in Turkey, as it is worldwide. Current data will contribute to a better understanding of the epidemiologic dimension of COPD in our country.

## 1. Introduction

Chronic obstructive pulmonary disease (COPD) is a preventable and treatable major public health problem. It is often associated with a history of cigarette smoking and is the primary contributor to mortality caused by chronic lower respiratory diseases, which is the third leading cause of death worldwide [1]. Although it is an important and increasing global cause of morbidity and mortality, there is still insufficient knowledge about COPD, and it is not adequately diagnosed. The prevalence and mortality of COPD are expected to increase, particularly in developing countries such as Turkey, and the natural history of COPD in the community is largely unknown, including in Turkey.

Accurate epidemiologic information is important in chronic diseases, including COPD, for several reasons: documenting of the impact of COPD on quality of life and costs, helping to inform public health planning, and efficiently planning future healthcare [2]. 

Turkey is a developing Eurasian country; it does not yet have a nation-wide medical registry system solely devoted to collecting data about various chronic diseases such as COPD. The present study aimed to evaluate the epidemiologic characteristics of COPD (prevalence, incidence, and mortality) within the entire Turkish population over a period of 5 years using the registry of the Social Security System, which covers 98.6% of the population, and in this way provide a useful and reliable resource for epidemiologic data about the Turkish population [3].

## 2. Materials and methods 

The data of this retrospective study were sourced exclusively from the Social Security System of Turkey in the scope of a cooperation protocol with the Social Security Institution. All data were extracted from the Oracle database using the Tool for Oracle Application Developers (TOAD) 9.6.0.27 program. 

The Social Security System of Turkey collects and collates data regarding all hospital admissions and discharge registries in Turkey on an individual basis. Since 1996, the tenth revision of the World Health Organization International Statistical Classification of Diseases and Related Health Problems (ICD-10) has been used to code hospital admissions and discharges in Turkey [4]. Individuals aged over 18 years were identified as having physician-diagnosed COPD if they had 1 COPD hospitalization and/or 1 COPD ambulatory care claim as indicated by the following codes in the registry of the Social Security System: J44.0-J44.9. Data related with the population of Turkey during the study were acquired from the website of the Turkish Institute of Statistics [5]. 

Incident cases were defined as individuals who had no COPD-related healthcare presentations in the previous year. To calculate the annual incidence rates from 2012 to 2016, we divided the number of individuals who developed COPD in a given year by the number of individuals at risk for COPD (the total population minus the number of people with prevalent COPD in the previous year). Prevalent cases were defined as individuals who had COPD diagnoses with ICD codes J44.0-J44.9 and remained in the study population until they died. To calculate the annual prevalence rates of COPD in the population from 2012 to 2016, we divided the number of patients with COPD who were alive at the end of each year by the census population estimate of that same year. COPD deaths were defined as individuals who had COPD diagnoses and died of any cause during the study period (all-cause mortality). We calculated the annual mortality rate from 2012 to 2016 by dividing the annual number of deaths in people with COPD by the number of individuals with COPD in each year.

In Turkey, pulmonologists initiate COPD treatment according to regulations. Other general or family practitioners usually refer patients who they suspect of having COPD to pulmonologists. There is no referral system in Turkey [6,7]. We used the criterion of a fixed postbronchodilator ratio of forced expiratory flow in 1 s (FEV1)/forced vital capacity (FVC) of less than 0.7 as the primary indicator of airflow obstruction, as proposed by the Global Initiative for Chronic Obstructive Lung Disease (GOLD) in Turkey [8]. 

**Figure 1:**
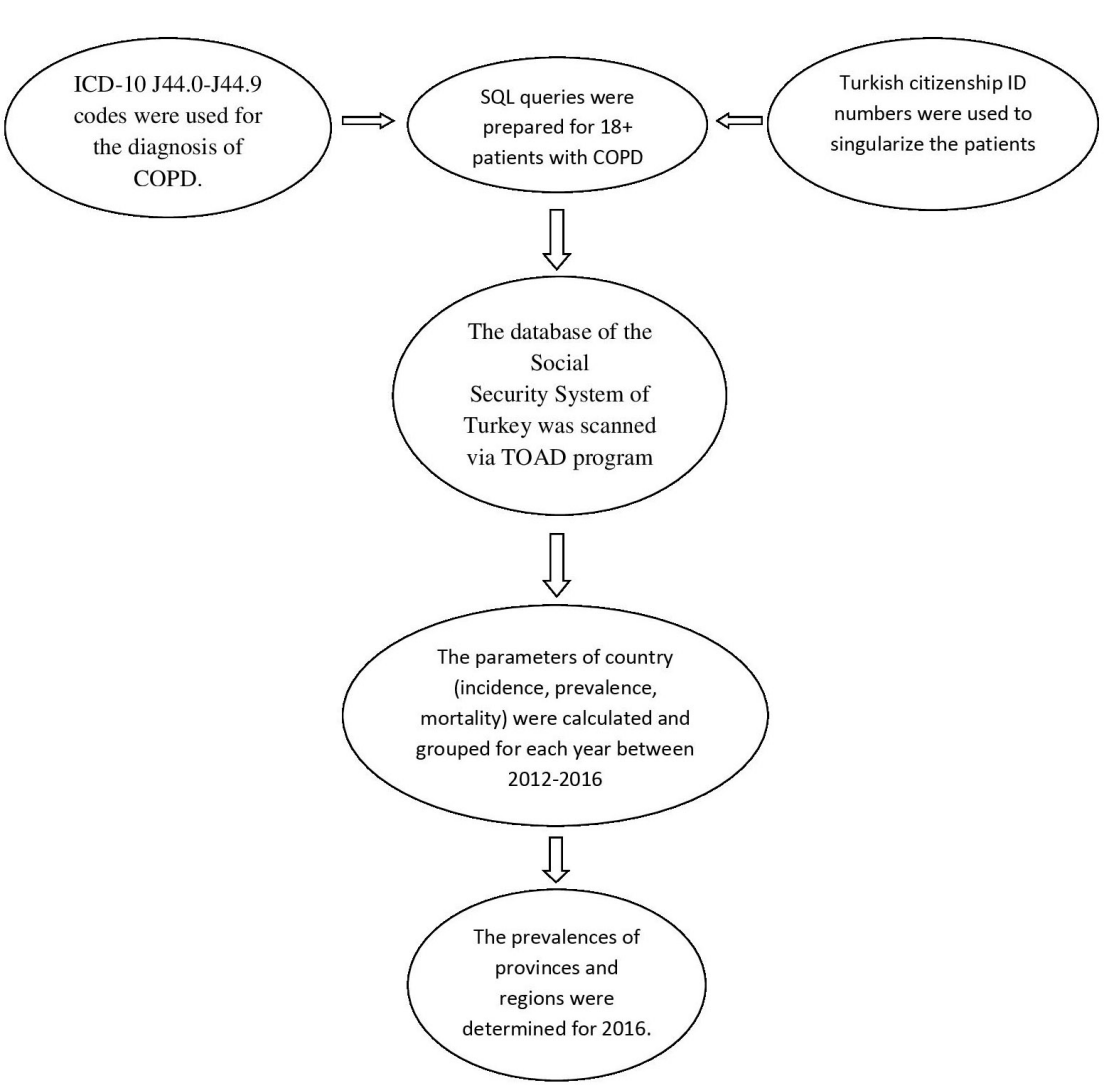
Flow chart of methodology.

In the current analysis, we report the overall prevalence, incidence, and mortality data in all physician-diagnosed individuals with COPD from 2012 to 2016, but the prevalence data of cities and regions are limited up to 2016. The methodology is presented as flow chart (Figure 1).

The present study was approved by the Ethics Committee and Institutional Review Board of Dr. Abdurrahman Yurtaslan Oncology Hospital (November 14, 2018; 2018-11/136). 

SAS Enterprise Guide 5.1 (SAS Institute Inc. Cary, NC, USA) was used for statistical analysis of the collated data. Graphs were drawn using Graphpad Prism 6.01 (GraphPad Software Inc., La Jolla, CA, USA). Continuous variables are expressed as numbers or percentages where appropriate. The Mann–Whitney U-test and χ2 tests were used for comparisons; 2-tailed P-values of <0.05 were accepted as statistically significant.

## 3. Results

**Figure 2:**
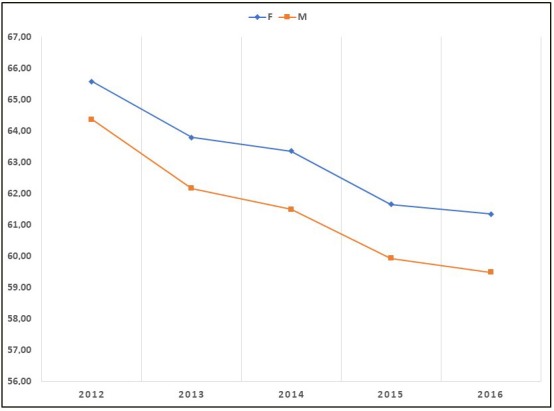
Average age of newly-diagnosed patients with COPD in Turkey, from 2012 to 2016.

In 2012, 466,457 COPD patients with a mean age of 65.59 ± 16.63 years were diagnosed as having COPD for the first time. In 2016, 359,392 patients with a mean age of 60.25 ± 15.45 years were newly diagnosed as having COPD (Table 1). There were 3,434,262 cases of COPD (56.2% men) in Turkey in 2016. The overall mean age of the patients was 61.62 ± 14.76 years. The mean age of men and women was 61.16 ± 14.24 years and 62.21± 15.38 years, respectively; 31.3% of the patients were aged over 70 years. From 2012 to 2016, the age of diagnosis decreased both in males and females, and women were diagnosed at a later age compared with men (Figure 2). 

**Table 1:** Demographic characteristics of newly-diagnosed patients with COPD.

Years	Sex	Newly-diagnosedpatients with COPD (n)	Mean age ± SD	Total(n)	Mean age ± SD
2012	F	189,704	65.59 ± 16.63	466,457	64.87 ± 15.95	M	276,753	64.37 ± 15.00
	2013	F	172,331			63.80 ± 16.27	419,414	62.84 ± 15.35	M	247,083	62.17 ± 14.65
2014		F	162,916	63.36 ± 16.24	395,351	62.26 ± 15.38			M	232,435	61.50 ± 14.70
	2015	F	161,858	61.66 ± 16.39			391,542	60.64 ± 15.49	M	229,684	59.93 ± 14.77
2016		F	149,317	61.35 ± 16.37	359,392	60.25 ± 15.45			M	210,075	59.48 ± 14.71

COPD: Chronic obstructive pulmonary disease; SD: Standard deviation; F: Female; M: Male

**Figure 3:**
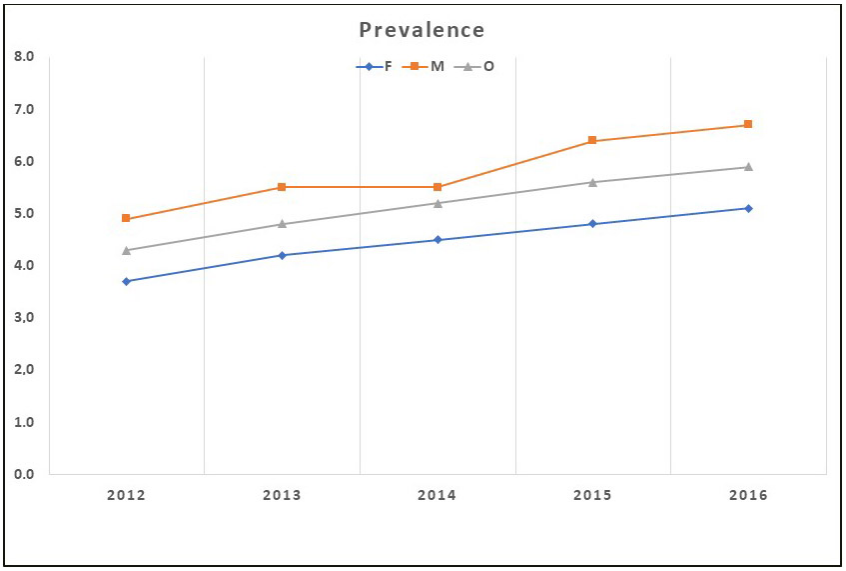
Annual overall and sex-stratified physician-diagnosed COPD prevalence rates in Turkey, from 2012 to 2016.

From 2012 to 2016, the overall annual prevalence rates of physician-diagnosed COPD rose from 4.3% to 5.8%, which was a 35.0% relative increase (P for trend < 0.05). In the same period, the annual prevalence rates of physician-diagnosed COPD in women rose from 3.7% to 5.1% (38%, P for trend < 0.05). This rate rose from 4.9% to 6.7% (37%, P for trend < 0.01) in men. The prevalence rate increase was the same in both sexes. COPD rates were higher in men than in women at any year interval, and there was an increasing trend in COPD prevalence rates among both sexes (Figure 3).

**Figure 4:**
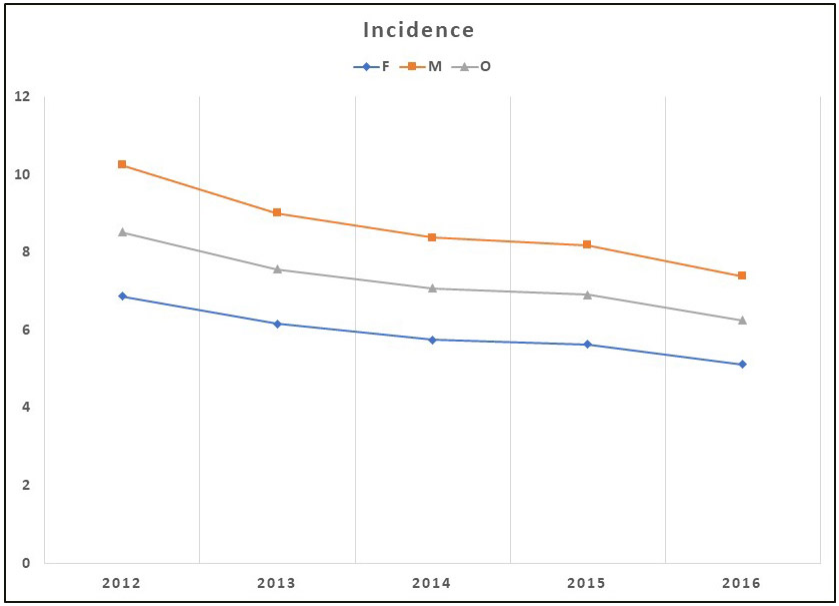
Annual overall and sex-stratified physician-diagnosed COPD incidence rates in Turkey, from 2012 to 2016.

During the study period, the overall incidence rate of physician-diagnosed COPD decreased from 8.5 per 1000 adults in 2012 to 6.3 per 1000 adults in 2016, representing a relative decrease of 26.6% (P < 0.001). In the same period, the annual incidence rates of physician-diagnosed COPD in women decreased from 6.9 per 1000 adults to 5.1 per 1000 adults (25.4%, P for trend < 0.05). This rate decreased from 10.2 per 1000 adults to 7.4 per 1000 adults (27.9%, P for trend < 0.001) in men. Incidence rates were higher among men than in women at any year interval, and incidence rates had a decreasing trend in both sexes (Figure 4).

**Figure 5:**
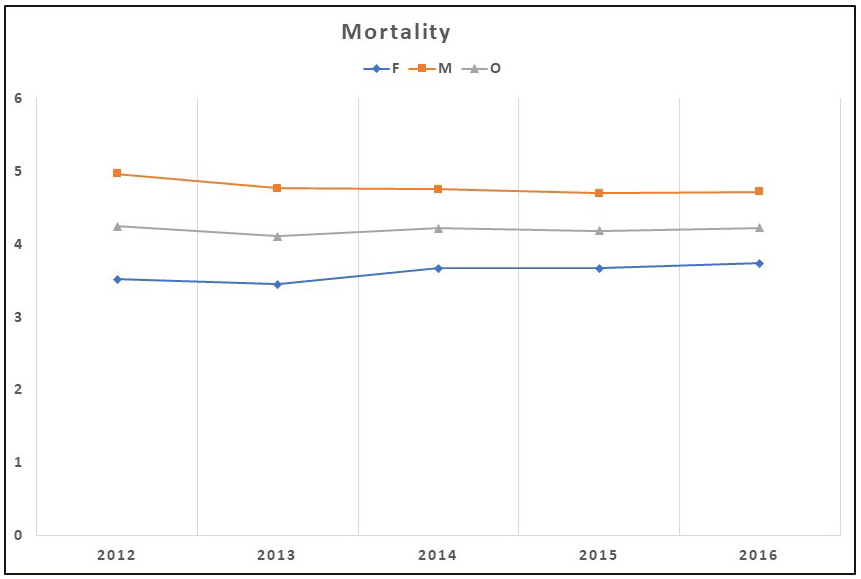
Annual overall and sex-stratified physician-diagnosed COPD mortality rates in Turkey, from 2012 to 2016.

Mortality rates were significantly higher in men than in women at any year interval. The mortality rate was 3.5% in 2012 and 3.7% in 2016 in women (P > 0.05), and 5% in 2012 and 4.7% in 2016 in men (P > 0.05). There was no significant difference between the overall mortality rate in 2012 and 2016 (4.3% vs. 4.2%) (Figure 5). 

**Figure 6:**
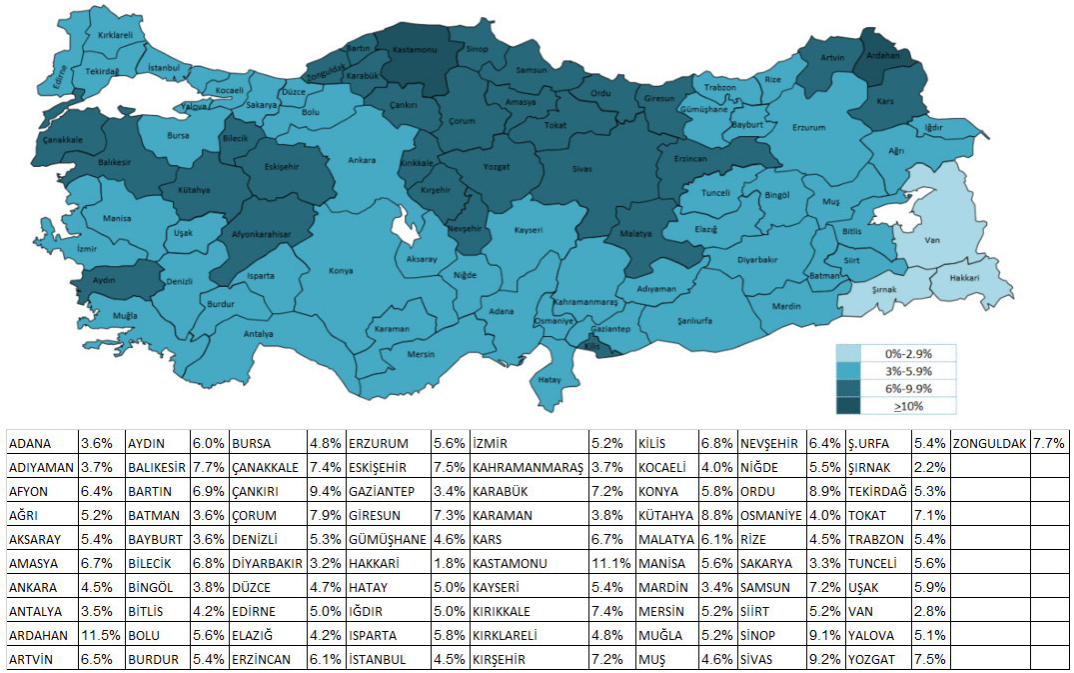
Prevalence of physician-diagnosed COPD by city in Turkey, in 2016.

The mean prevalence by region was 5.26% (range, 3.79%–7.65%). The Black Sea region had the highest COPD prevalence (Table 2). The prevalence of COPD varied considerably by city: 9.4%–11.5% in Ardahan, Kastamonu, and Çankırı, and 1.8%– 2.79% in Hakkari, Şırnak, and Van. The median prevalence by city was 5.68% (range, 1.8%–11.5%) (Figure 6).

**Table 2:** The prevalence rates by region in Turkey in 2016.

Region	Prevalence (%)
Black Sea	7.65
Central Anatolia	6.13
Aegean	5.68
Eastern Anatolia	4.84
Marmara	4.48
Mediterranean	4.23
Southeast Anatolia	3.79

## 4. Discussion

This population-based study reported overall prevalence, incidence, and mortality data in all individuals with physician-diagnosed COPD over a 5-year period in Turkey. We found that the prevalence of COPD increased by 35.0%, the incidence of COPD decreased by 26.6%, and the overall all-cause mortality of individuals with COPD was 4.2% between 2012 and 2016 in Turkey. Most of the increase in prevalence was borne by men. We also found the incidence of COPD to be decreasing (more pronounced in men than in women) and all-cause mortality stability, suggesting that COPD management strategies were having a beneficial effect.

Prevalence is dependent on incidence and duration of a disease. The prevalence of COPD can be evaluated through different approaches: physician-diagnosed COPD prevalence, the prevalence of respiratory symptoms revealed in questionnaires, and the prevalence of spirometry and airflow limitation. Also, it is difficult to compare published studies because of the great variability that exists in terms of the designs, diagnostic criteria, and age ranges used. Rycroft et al. highlighted this situation in their review, showing that the prevalence ranged from 0.2% to 37% between different countries [9]. For example, in China, Zhong et al. interviewed participants using a standardized questionnaire and performed spirometry on all eligible participants to determine the COPD prevalence among citizens aged 40 years or older, and found the overall prevalence of COPD was 8.2% (men, 12.4%; women, 5.1%) [10]. In India, an average prevalence of 3.5% was reported in a study with a large population (INSEARCH) undertaken at 16 different centers in the country [11]. In the PLATINO study, individuals were invited to answer a questionnaire and underwent anthropometry and spirometry. The prevalence of COPD ranged from 7.8% to 20% in 5 major Latin American cities [12]. In the BOLD study, the prevalence of stage II or higher COPD was 10.1% overall, 11.8% for men, and 8.5% for women [13]. However, in the results of screening studies, the prevalence of physician-diagnosed COPD was different. In Austria, Schirnhofer et al. surveyed a sex-stratified, population-based sample of 2200 adults ≥40 years of age, and they found that the overall prevalence of mild COPD was 26.1%, whereas the prevalence of moderate or severe COPD was 10.7%. Furthermore, a doctor diagnosis of COPD was reported by only 5.6% of participants [14]. In the United Kingdom, from 1990 to 1997 the annual prevalence rates of physician-diagnosed COPD in women rose from 0.80% to 1.36%, and 1.3% to 1.6% in men. The prevalence rate increased by 68.7% in women compared with 25.3% in men, and COPD rates were higher in men than in women at any age interval [15]. Our prevalence estimates of COPD are consistent with these studies. We found that the overall prevalence rates of physician-diagnosed COPD rose from 4.3% to 5.8%, in women it rose from 3.7% to 5.1%, and in men it increased from 4.9% to 6.7%. COPD rates were higher in men than in women at any year interval. 

To the best of our knowledge, although a number of regional reports are available, there have been no previous large-scale, population-based studies of trends in COPD prevalence, incidence, and mortality in Turkey. COPD prevalence, risk factors, and burden of disease were evaluated in the BOLD study in Adana by Kocabaş et al. between December 2003 and January 2004. The preliminary results of this study showed that COPD prevalence was about 19.1% in subjects aged over 40 years in Adana [16]. Günen et al. evaluated the prevalence of COPD in a population randomly selected from urban and rural regions of Malatya. Through a respiratory questionnaire and postbronchodilator spirometry performed in 1160 participants aged over 18 years, COPD prevalence was found as 6.9%. The prevalence of COPD was 18.1% in current smokers, and 4.5% among young smokers [6]. Deveci et al. evaluated 1206 participants aged >18 years using a respiratory questionnaire and spirometry in order to determine the prevalence of COPD in Elazığ. The prevalence of COPD in those aged ≥18 years was 4.5% (female 2.5%; male 6%), and the prevalence in participants aged ≥45 years was 11.5% (female 5.9%; male 15.1%) [17]. Çetinkaya et al. performed a crosssectional survey with 1023 subjects aged 20–83 years, and they found the prevalence was higher in males (17.8%) than in females (10.0%) [18]. Our prevalence estimates of COPD are lower than those found in these screening studies. These differences can be explained by the fact that the COPD prevalence in our study was based on symptomatic patients who presented to a physician and not on broad-based spirometric findings or questionnaires. 

The incidence of COPD varies greatly between countries, but it is difficult to compare estimates because they are reported in different units and over different lengths of time. De Marco et al. found the incidence rate of COPD as 2.8 cases/1000/year in Italy [19]. In Sweden, the 10-year cumulative incidence of COPD was estimated at 8.2% with the British Thoracic Society criteria, and 13.5% with the GOLD criteria [20]. Although the incidence COPD has shown an increasing trend over the years, there has been an overall decrease in the last 10 years. Gershon et al. reported that the incidence of COPD decreased in Ontario from 11.8 per 1000 adults in 1996 to 8.5 per 1000 adults in 2007, representing a relative decrease of 28.3% [21]. Also in our study, the overall incidence rate of physician-diagnosed COPD decreased from 8.5 per 1000 adults in 2012 to 6.3 per 1000 adults in 2016, representing a 26.6% decrease. The incidence of COPD also varied between the sexes. In the same study by Gershon et al., the incidence of COPD was demonstrated to be decreasing in men more notably than in women. The incidence rates had a decreasing trend in both sexes [21]. In Australia, the incidence of COPD decreased in men between 1998 and 2003, but increased in women [22]. We found the annual incidence rates of physician-diagnosed COPD in women decreased from 6.9 per 1000 adults to 5.1 per 1000 adults (25.4%) between 2012 and 2016, and from 10.2 per 1000 adults to 7.4 per 1000 adults (27.9%) in men. Our incidence rates also had a decreasing trend in both sexes. All these studies and ours suggest improvements in COPD management. 

The real extent and impact of COPD on mortality in the general population is incompletely known. Similar to prevalence and incidence studies, the reported mortality rates among patients with COPD and length of follow-up differ, which results in difficulties when comparing studies. In Sweden, the OLIN COPD study longitudinally compared patients with and without COPD, and the mortality rate was significantly higher among subjects with COPD (5.1% vs. 3.0%) [23]. In the study by Camp et al., men had a higher prevalence (4.7% vs. 4.0%) and a higher all-cause mortality rate (5.4% vs. 4.1%) than women in 2003/2004. The authors also found a trend of decreasing age-standardized mortality rates from 1992/1993 to 2000/2001, followed by a stable mortality rate from 2001 onward for both men and women with COPD in Canada [24]. Similar to these results, Gershan et al. found that the all-cause mortality rate decreased from 5.7% in 1996 to 4.3% in 2007, representing a 24.0% relative decrease. This decrease was greater in men than in women. Mortality rates were significantly higher in men at any year interval [21]. We found mortality rates of 3.5% in 2012 and 3.7% in 2016 in women, and 5% in 2012 and 4.7% in 2016 in men, and the overall mortality rates in 2012 and 2016 were 4.3% and 4.2%, respectively. The mortality rate in women with COPD increased minimally but then plateaued, as in men. Decreasing COPD mortality rates are also consistent with improvements in COPD management. 

When Turkey is examined in terms of regional distribution of prevalence, it can be seen that the prevalence is heterogeneous. The mean prevalence by region was 5.26%, and by city it was 5.68%. The Black Sea region had the highest COPD prevalence. This may be related to the presence of an elderly population in the Black Sea region [25]. In the case of smoking in our country, men between the ages of 35 and 44 years constitute the age group that smokes the most tobacco products, at 50.6% [5]. According to these data, the prevalence of COPD in Turkey would be expected to be higher than we found. The case data were exclusively sourced from the Social Security System. We used physician-diagnosed COPD and, therefore, underestimated the prevalence. Additional research is needed to determine the underlying causes of geographic distributions, which might be related to geographic variations in other factors, including diagnostic practices, access to effective health interventions, cigarette smoking, and occupational and environmental exposures.

This study has a series of advantages, the principal of which is that it covered the entire population of Turkey because the Social Security System covers 98.6% of the population. Accordingly, there should be no selection bias present. Furthermore, this is the first large, population-based study to examine trends in COPD prevalence, incidence, all-cause mortality, and regional trends. A number of limitations of this analysis must be mentioned. The main limitation is that we used physician-diagnosed COPD, which is only the tip of the iceberg and may underestimate the true prevalence. Secondly, in daily practice, the actual cause of death remains in the background or multiple diagnoses can be made, so the mortality rate is the rate of all-cause mortality. Thirdly, this is a descriptive study and does not address factors such as environmental, occupational, socioeconomic, and healthcare-related factors in determining COPD morbidity, mortality, and health service use. By providing a variety of data such as demographic data in the registration information in the Social Security System database, information about the factors affecting environmental and occupational exposures in COPD development can be obtained.

In conclusion, differences in the results between countries may be due to differences in notification systems, as well as differences in smoking behaviors in countries, environmental factors, infections, and the distribution of genetic factors. Our results suggest that COPD management strategies are having a benefit in Turkey. According to these results, COPD is a much larger health problem in Turkey, but the actual extent of COPD will only emerge when nondiagnosed candidates who self‑report good health are included in investigations. We hope that these results will stimulate healthcare providers and increase the attention of policy-makers to take action towards this important disease. We hope our results will have positive effects on COPD prevention and management strategies. Finally, we think that the findings of our study will provide a basis for further investigations.

## Acknowledgement

All authors have no conflict of interest or financial disclosure. This manuscript has not been published previously or is not under consideration for publication elsewhere. This study was presented by Nilgün Yılmaz Demirci as oral presentation in Turkish Respiratory Society, RESPIRATION 2018 in Antalya on October 13–16, 2018.

## References

[ref0] (08). The top 10 causes of death.

[ref1] (2005). The burden of obstructive lung disease initiative (BOLD): rationale and design. Journal of Chronic Obstructive Pulmonary Disease.

[ref2] (2019). -2019 Stratejik Planı. pdf [accessed 05.08.

[ref3] (1996). Manual of the International Statistical Classification of Diseases, Injuries and Causes of Death.

[ref4] tr [accessed 20.03.

[ref5] (2008). Prevalence of COPD: first epidemiological study of a large region in Turkey. European Journal of Internal Medicine.

[ref6] (2015). Categorization of COPD patients in Turkey via GOLD 2013 strategy document: ALPHABET study. International Journal of Chronic Obstructive Pulmonary Disease.

[ref7] (2011). Initiative for Chronic Obstructive Lung Disease (GOLD) Global strategy for the diagnosis, management, and prevention of chronic obstructive pulmonary disease. (Accessed November.

[ref8] (2012). Epidemiology of chronic obstructive pulmonary disease: a literature review. International Journal of Chronic Obstructive Pulmonary Disease.

[ref9] (2007). Prevalence of chronic obstructive pulmonary disease in China. The American Journal of Respiratory and Critical Care Medicine.

[ref10] (2012). Indian study on epidemiology of asthma, respiratory symptoms and chronic bronchitis in adults (INSEARCH). International Journal of Tuberculosis and Lung Disease.

[ref11] (2005). Chronic obstructive pulmonary disease in five Latin American cities (the PLATINO study): a prevalence study. Lancet.

[ref12] (2007). BOLD Collaborative Research Group International variation in the prevalence of COPD (The BOLD Study): A population‑based prevalence study. Lancet.

[ref13] (2007). COPD prevalence in Salzburg, Austria: Results from the burden of obstructive lung disease (BOLD) study. Chest.

[ref14] (2000). Recent trends in physician diagnosed COPD in women and men in the UK. Thorax.

[ref15] (2006). Prevalence of COPD in Adana, Turkey (BOLD-Turkey study). Proceedings of the American Thoracic Society.

[ref16] (2011). The prevalence of chronic obstructive pulmonary disease in Elazig, Eastern Turkey. European Journal of Internal Medicine.

[ref17] (2000). Prevalence of chronic bronchitis and associated risk factors in a rural area of Kayseri, Central Anatolia, Turkey. Monaldi Archives for Chest Disease.

[ref18] (2007). Incidence of chronic obstructive pulmonary disease in a cohort of young adults according to the presence of chronic cough and phlegm. The American Journal of Respiratory and Critical Care Medicine.

[ref19] (2005). Ten-year cumulative incidence of COPD and risk factors for incident disease in a symptomatic cohort. Chest.

[ref20] (1996). Trends in chronic obstructive pulmonary disease prevalence, incidence, and. Archives of Internal Medicine.

[ref21] (2009). Trends in COPD mortality and hospitalizations in countries and regions of Asia-Pacific. Respirology.

[ref22] (2008). The obstructive lung disease in northern Sweden chronic obstructive pulmonary disease study: design, the first year participation and mortality. Clinical Respiratory Journal.

[ref23] (2008). The sex factor: epidemiology and management of chronic obstructive pulmonary disease in British Columbia. Canadian Respiratory Journal.

